# Migrant Health Burden: Where Do We Stand?

**DOI:** 10.3390/ijerph17093004

**Published:** 2020-04-26

**Authors:** Laura Spagnoli, Antoine Flahault, Pietro Ferrara

**Affiliations:** 1 Health Authority of Verbano-Cusio-Ossola, Via Mazzini 117, 28887 Omegna (VB), Italy; 2 Institute of Global Health, Faculty of Medicine, University of Geneva, CH-1202 Geneva, Switzerland; antoine.flahault@unige.ch; 3 Research Centre on Public Health, University of Milan-Bicocca, Via Pergolesi 33, 20900 Monza, Italy; p_ferrara@alice.it

**Keywords:** access to care, health disparities, international health, migrant health, minority health, refugees health, social determinants of health

## Abstract

This Special Issue of the *International Journal of Environmental Research and Public Health* contains a collection of extended papers that describe many important aspects of the “migrant health burden” and focus on new realities and solutions in the healthcare of migrants and refugees.

**Foreword**

Migration, constituting approximately 258 million international refugees and migrants worldwide [1], is a social determinant of health. As a consequence of this geographic dispersion through the movement of people, growing challenges are emerging to health systems and policies that will require responses at national and international levels [2].

Migrants face high barriers in accessing essential healthcare services due to multiple factors, ranging from difficulties in communication, irregular status, and lack of migrant-inclusive health policies to inaccessibility of services [3].

This Special Issue of the *International Journal of Environmental Research and Public Health* provides an insight into fundamental research in the migrant health field. A significant collection of contributions and studies has been presented. Each of them offers important insights into the emerging challenges of migrant care and suggests the future solutions to be addressed, as well as the baseline points for further research on these topics. The proposed articles cover, indeed, a wide range of thought-provoking themes.

Combes et al. [4] studied the connection between self-perceived health and socio-economic and demographic factors and length of stay amongst migrants and asylum seekers seen in Médecins du Monde free clinics across different European countries. One of the main findings of this study was that asylum seekers are in worse health than other documented patients. On the other hand, an effect of housing and job markets was found, with migrants having accommodation or a job in better health compared with those who did not. Thus, the authors highlighted that stable and safe accommodations and facilitating access to the job market for migrants are essential conditions for their health, also in line with the International Covenant on Civil and Political Rights and the European Convention on Human Rights. 

In the cross-sectional study by Leiler et al. [5], data on prevalence of distress severity and suicidal ideation were analyzed in a sample of asylum seekers in Sweden. A clear but not strong association between severity of distress and suicidal ideation actually exists. Even though the utility of suicidal ideation as a test for later suicide is limited by a modest sensitivity [6], the suicide risk among asylees and refugees cannot be underestimated. From this standpoint, it should be mentioned that routine mental health screening for migrants and refugees is an extremely imperative instrument upon arrival to the country of residence. 

Mental health is also the focus of the article by Espinoza-Castro et al. [7]. It suggests that the lack of privacy, individuals’ powerlessness to have control over their living and working conditions, length of stay, and work overload were associated with major depressive syndrome among Spanish-speaking au pairs in Germany. 

The lesson that can we learn from Castañeda’s study [8], meanwhile, is that the presence of large migrant communities—often also ethnically distinct from the resident population in terms of life habits—that have formed across certain more attractive areas commits the attention of public health to changes in lifestyle habits (e.g., eating habits, the consumption of alcohol and tobacco, etc.) and health promotion in these specific populations.

A short communication of pilot, but significant, results completes the Special Issue. Here, Hjern and Kling addressed the issue of healthcare needs in school-age refugee children in the school system in Malmö (Sweden), identifying main needs for dental and mental healthcare, as well as for care for disabilities and chronic disorders [9]. In this study, the authors found a high prevalence of mental health problems amongst refugee children, underlining—very much in line with other studies of this Special Issue—how mental health (and well-being) represent one of the major problems pending in migrants’ healthcare. Thus, two other important aspects come from Hjern and Kling’s article. Indeed, one of the most relevant challenges in the healthcare of migrants is the lack of documentation about their immunization status and vaccinations, and this may tighten up inequalities in access to health services in the countries where migrants live [10]. The second point is about the role that school contexts may play in promoting the health of children, calling for school-based programs for the health assessment and prevention of health problems in refugees and migrants.

Last, but not least, in his editorial, Pietro Ferrara highlights that little or nothing has been studied about the measurement of the economic burden of disease in migrants and refugees, despite the importance of this type of assessment for the continuous improvement of smoother and faster reallocation of resources in healthcare systems. This publication therefore focuses on the need for further investigation of the economic aspects of health and social care on the migrant population, also considering the ability to affect economic growth of hosting countries [11].

In a nutshell, the route towards the full addressing of the demand for migrants’ and refugees’ healthcare is long and arduous, but this Special Issue shows small but good progress and provides some starting points for discussion and strategies for addressing the emerging challenges [4,5,7,8,9,10,11]. We, as guest editors, hope that this Special Issue may serve as a stimulus for those who are interested in or involved with the discussion of challenges, opportunities, and solutions to overcome boundaries that affect the healthcare of migrants and refugees.
